# Supplementary material for: Venomix: a simple bioinformatic pipeline for identifying and characterizing toxin gene candidates from transcriptomic data
Source: PeerJ. 2018 Jul 31;6:e5361. doi: 10.7717/peerj.5361 (PMC6074769; doi:10.7717/peerj.5361)
Supplement: Supplemental Information 6 [file peerj-06-5361-s006.gz › FinalOutput_1E-6/Galactose-specific_lectin_nattectin_1/finaltree.pdf]

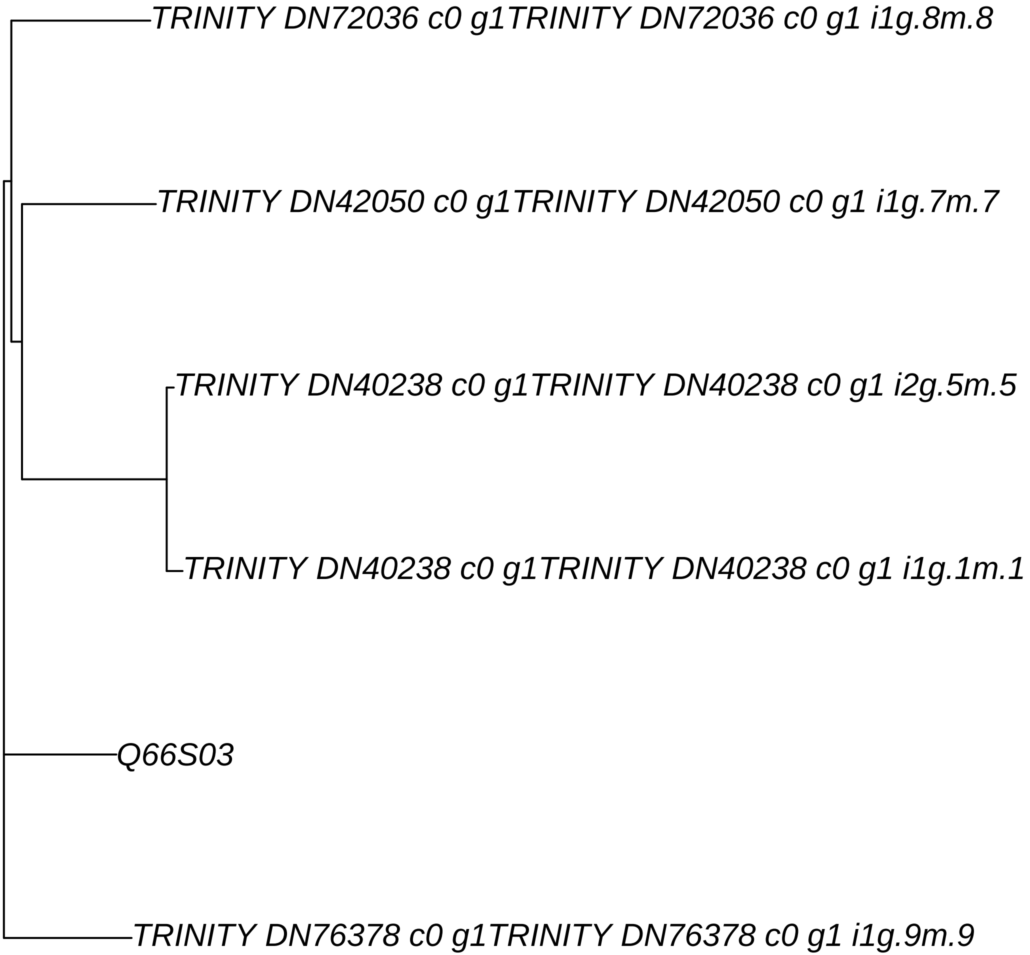
TRINITY DN72036 c0 g1TRINITY DN72036 c0 g1 i1g.8m.8
TRINITY DN42050 c0 g1TRINITY DN42050 c0 g1 i1g.7m.7
TRINITY DN40238 c0 g1TRINITY DN40238 c0 g1 i2g.5m.5
TRINITY DN40238 c0 g1TRINITY DN40238 c0 g1 i1g.1m.1
Q66S03
TRINITY DN76378 c0 g1TRINITY DN76378 c0 g1 i1g.9m.9
